# THE USE OF SPECIALIST HEALTH CARE IN NURSING HOMES AS AN INDICATOR FOR QUALITY OF CARE

**DOI:** 10.1093/geroni/igz038.286

**Published:** 2019-11-08

**Authors:** Karin Wolf-Ostermann, Karin Wolf-Ostermann, Annika Schmidt, Daniel Gand, Ansgar Gerhardus, Heinz Rothgang

**Affiliations:** 1 University of Bremen, Bremen, Germany; 2 University of Bremen, Bremen, Bremen, Germany

## Abstract

Needs-based medical care is essential for maintaining and promoting health and quality of life in long-term care. But still knowledge of medical needs and of needs-based medical care of nursing home (NH) residents is limited. However, individual studies, primarily based on secondary data, show that NH residents have significantly less contact with specialists than community-dwelling elderly. The aim of the study is therefore to gain knowledge about medical needs of NH residents as well as about the use of (specialist) medical care especially with respect to vision, hearing and oral health. Besides sociodemographic and care-related data we evaluated health status and utilization of medical care by means of standardized assessments for n=500 NH-residents. Results show that there are inequalities in (specialist) medical care: More experienced knowledge about unmet medical care needs will be used to generate ideas on how to improve the provision of medical care in nursing homes.

